# The relative efficiency of Iranian’s rural traffic police: a three-stage DEA model

**DOI:** 10.1186/s12889-017-4780-z

**Published:** 2017-10-13

**Authors:** Habibollah Rahimi, Hamid Soori, Seyed Saeed Hashemi Nazari, Seyed Abbas Motevalian, Adel Azar, Eskandar Momeni, Mehdi Javartani

**Affiliations:** 1 Trauma Research Center, Kashan University of Medical Sciences, Kashan, IR Iran; 2 Department of Epidemiology, Safety Promotion and Injury Prevention Research Center, School of Public Health, Shahid Beheshti University of Medical Sciences, Tehran, IR Iran; 3 Department of Epidemiology, School of Health, Iran University of Medical Sciences, Tehran, IR Iran; 4 Department of Management, Faculty of Management and Economics, Tarbiat Modares University, Tehran, Iran; 5 Traffic Police of Islamic Republic of Iran, Tehran, Iran; 6 Department of Biostatistics, Faculty of Medical Sciences, Tarbiat Modares University, Tehran, Iran

**Keywords:** Traffic police, Efficiency, Data envelopment analysis

## Abstract

**Background:**

Road traffic Injuries (RTIs) as a health problem imposes governments to implement different interventions. Target achievement in this issue required effective and efficient measures. Efficiency evaluation of traffic police as one of the responsible administrators is necessary for resource management. Therefore, this study conducted to measure Iran’s rural traffic police efficiency.

**Methods:**

This was an ecological study. To obtain pure efficiency score, three-stage DEA model was conducted with seven inputs and three output variables. At the first stage, crude efficiency score was measured with BCC-O model. Next, to extract the effects of socioeconomic, demographic, traffic count and road infrastructure as the environmental variables and statistical noise, the Stochastic Frontier Analysis (SFA) model was applied and the output values were modified according to similar environment and statistical noise conditions. Then, the pure efficiency score was measured using modified outputs and BCC-O model.

**Results:**

In total, the efficiency score of 198 police stations from 24 provinces of 31 provinces were measured. The annual means (standard deviation) of damage, injury and fatal accidents were 247.7 (258.4), 184.9 (176.9), and 28.7 (19.5), respectively. Input averages were 5.9 (3.0) patrol teams, 0.5% (0.2) manpower proportions, 7.5 (2.9) patrol cars, 0.5 (1.3) motorcycles, 77,279.1 (46,794.7) penalties, 90.9 (2.8) cultural and educational activity score, 0.7 (2.4) speed cameras. The SFA model showed non-significant differences between police station performances and the most differences attributed to the environmental and random error. One-way main road, by road, traffic count and the number of household owning motorcycle had significant positive relations with inefficiency score. The length of freeway/highway and literacy rate variables had negative relations, significantly. Pure efficiency score was with mean of 0.95 and SD of 0.09.

**Conclusions:**

Iran’s traffic police has potential opportunity to reduce RTIs. Adjusting police performance with environmental conditions is necessary. Capability of DEA method in setting quantitative targets for every station induces motivation for managers to reduce RTIs. Repetition of this study is recommended, annually.

## Background

Road traffic injuries (RTIs), are the 9th cause of burden of disease (DALYs) in the world and it has been predicted to raise to the third order in 2030 [17]. To mitigate this problem, the governments have launched several safety traffic polices as well as some measures.

Most conducted interventions targeted to change driving behaviors while most of them are performed by the traffic police. Target achievements in this issue depend on selecting effective measures and accessing to consistent resources. In the field of Epidemiology, these issues are about the effectiveness and efficiency measurements. In encyclopedia of Epidemiology the effectiveness is defined as; "the extent to which a particular health technology (medical, device, drug, procedure, health program or health service, including intervention) does what it is intended to do (i.e. it leads to a beneficial health outcome or result) when it is provided under clinical practice conditions or in the field" [8] and in dictionary of epidemiology efficiency is defined as; "the relationship between resources (capital and labor) and health outcome" [16].

The effectiveness of some of the traffic police interventions such as speed reduction, using seat belt or breath alcohol testing have been studied [12, 23, 25].

However, traffic police efficiency has not specifically been studied. In some studies, overall road safety has been measured. Hermans and et al., ranked road safety of 21 European countries using some indicators of driving behavior (percentage of using seatbelt, percentage of legal speed, driving below alcohol threshold) with some relevant infrastructure indicators as the input variables and numbers of injuries as well as deaths as the outputs [15]. In the above mentioned study, overall road safety was measured. In another study the trend of road safety efficiency of US states was investigated [13] in which traffic volume, road maintenance and safety expenditure were used as the input variables and death caused by RTIs as the output variable. Similar to the study done by Hermans et al., the overall road safety efficiency was measured. Just Aristovnik et al. measured the efficiency of criminal and traffic performance of police in Slovenia [3, 4].

In all the above studies, the efficiency was measured using Data Envelopment Analysis (DEA) method. This method was originated by Charnes, Cooper and Rhodes in 1978 and measures relative efficiency of decision making units (DMUs) [9]. Decision making unit (DMU) refers to an educational, health, military or some other administrative units with the authority in producing outputs using inputs. Applying this method, an efficiency score (ranged 0–1) is assigned to every DMU. DMUs with the efficiency of 1 are the benchmark of DMUs with the efficiency score of less than 1. In addition, this method determines an increase in efficiency score whose inputs should be decreased or whose outputs should be increased through benchmarking from peer DMUs. Another application of this method is determining short time quantitative targets for every DMU. This is based on available inputs for every DMU. In RTIs issue, setting targets induces incentive for policy makers and managers for RTIs reduction [24].

Iran, with high rate of RTIs, has achieved successes in reducing such public health problem. According to a national analysis, the trend of death rate due to RTIs declined from 38 to 31 per 100,000 from 2004 to 2011. Whilst, in this period, registered vehicles have increased [5]. This is as the result of various measures such as increasing law enforcement performed by the Iranian traffic police, entitled RAHVAR.

Taking into account the importance of traffic police, this study aimed at measuring the relative efficiency of local police stations of RAHVAR in rural areas of Iran, applying three- stage DEA method.

## Methods

### Efficiency measurement

In cases where there is an input and output, the efficiency score is simply obtained from the ratio of output to input. In occasions with more than input and output, it is required to assign weights to variables and to insert sum of weighted outputs to inputs. The weakness of this method refers to the disagreements between experts about values of assigned weights.

Due to some constraints and through solving mathematical programming, in DEA, as a nonparametric method, the optimum weights were assigned to inputs and outputs for every DMU. The assigned weights are obtained the maximum possible efficiency score of DMU. Having combined the weighted input and output variables, a virtual DMU is made that with respect to the input-output oriented approach, either uses minimum level of inputs whilst keeping outputs constant or produces maximum level of outputs whilst keeping inputs constant. The ratio of virtual DMU to considered DMU determines the efficiency score [10]. Selecting input/output-oriented approach refers to the possibility of reducing input usage or increasing output production. For instance, to measure the efficiency of hospitals, the outputs are the treated patients and cannot be changed, arbitrary. While it is possible to reduce input usage, man powers (doctors, nurses). In this case, it is preferred to apply input-oriented DEA model. For traffic police, the police stations try to reduce crashes as much as possible with the available resources. Therefore, it is suitable to conduct output-oriented DEA model.

In addition to input/output-oriented approaches, there are two CCR (Charnes, Cooper and Rhodes) and BCC (Banker, Charnes and Cooper) models [6, 9]. CCR model is based on constant return to scale (CRS) assumption. It means input augmentation produces output increment at the same ratio. The CCR-Output oriented (CCR-O) efficiency score is obtained by solving the below equations and constraints.

1 $$ {\displaystyle \begin{array}{l}\left(\mathrm{CCR}-{\mathrm{O}}_{\mathrm{o}}\right){\mathrm{max}}_{\upeta \kern0.1em \upmu}\kern2em \upeta \\ {}\kern0.5em \mathrm{subject}\  \mathrm{to}\ {\mathrm{x}}_{\mathrm{o}}-\mathrm{X}\upmu \ge 0\kern5em \\ {}\kern4.25em {\upeta \mathrm{y}}_{\mathrm{o}}-\mathrm{Y}\upmu \le 0\kern7.5em \\ {}\kern7em \upmu \ge 0\kern1.75em \end{array}} $$

Where (ɳ) is the efficiency score of CCR-O model, (μ) is the output and input weights, (o) is the number of DMU, (X) is the input variable, (Y) is the output variable, (x_o_) and (y_o_) are the input and output variables of under measurement DMU.

In BCC model, with respect to the variable return to scale assumption (VRS), input augmentation cannot increase the output as the same ratio. According to the scale size of DMU, the output increment could be more, less or equal to the input increment. This could be due to non-coordination between various sectors of DMU_s_ with big scale sizes or requiring minimum inputs to starting performance in DMU_s_ with small scale sizes. In this study the BCC-O model was applied to extract pure technical efficiency from scale efficiency [6].

The BCC-O model is solving by eq. (2).

2 $$ {\displaystyle \begin{array}{l}\left(\mathrm{BCC}-{\mathrm{O}}_{\mathrm{o}}\right){\mathrm{max}}_{\upeta \mathrm{B},\uplambda}{\upeta}_{\mathrm{B}}\\ {}\kern0.5em \mathrm{subject}\  \mathrm{to}\kern0.75em \mathrm{X}\uplambda \le {\mathrm{x}}_{\mathrm{o}}\\ {}\kern2.25em {\upeta}_{\mathrm{B}}{\mathrm{y}}_{\mathrm{o}}-\kern0.5em \mathrm{Y}\uplambda \le 0\kern4.5em \\ {}\kern4.75em \sum \uplambda =1\\ {}\kern5.75em \uplambda \ge 0\end{array}} $$

Where (ɳ_B_) is the efficiency score of BCC-O model, (λ) is the output and input weights, (o) is the number of DMU, (X) and (Y) are the input and output variable of DMU_s_, (x_o_) and (y_o_) are the input and output of the under measurement DMU [10]. The goal of solving eq. (2) is obtaining efficiency score aimed to increase output production as much as possible while keeping input values at the same level.

In eq. (2), the outcomes are desirable. Since in this study the outcomes were non- desirable (the number of crashes caused damage, injury and death), therefore, the values of outcomes were inversed with subtracting from maximum value. To avoid zero value of DMU(s) with maximum value, one is added to all values [1].

Subtracting (Yλ) from (ɳ_B_y_o_) in eq. (2) provides output slacks. It means, for inefficient DMUs with efficiency score less than one, it is possible to increase outputs to (ɳ_B_) times while keeping inputs at the same level. This capability of DEA model could provide opportunity to set quantitative targets for every inefficient DMU. These targets are determined with benchmarking from peer efficient unit(s). Since transformed value of non-desirable outcomes have been used in this study; hence, instead of adding slacks to the outcomes of inefficient DMU_s_ as the target, they are subtracted.

One of the limitation of DEA method refers to deterministic property. Therefore, the effect of statistical noise on efficiency score is not accounted. In other words, obtained efficiency score may be due to measurement errors, or sampling variations. Bootstrap has been proposed as a method to consider sampling variations and stability of measured efficiency score [22]. Since this study aimed to measure efficiency score of total Iran’s police stations, the sampling variations were not a problem. While measurement error remained as a limitation of DEA method in this study. To solve this problem and also to extract the effect of environmental factors on police efficiency (non-discretionary), a method should be adopted that treat both of them.

Using three-stage DEA model, the pure technical efficiency is obtained without environmental and random error effects [10]. At the first stage crude efficiency score is measured using the original inputs and outputs. Next, after standardization, the slacks of stage one are regressed to environmental variables with applying Stochastic Frontier Analysis (SFA) model [7]. See eq. (3).

3 $$ \frac{{\mathrm{s}}_{\mathrm{rj}}^{+}}{{\mathrm{y}}_{\mathrm{rj}}}={\upbeta}_{\mathrm{ro}}+\sum_{\mathrm{k}=1}^{\mathrm{K}}{\upbeta}_{\mathrm{rk}}{\mathrm{lnZ}}_{\mathrm{k}\mathrm{j}}+{\mathrm{v}}_{\mathrm{rj}}+{\upupsilon}_{\mathrm{rj}}\kern2.75em \left(\mathrm{r}=1,\dots, \mathrm{s}\right) $$

Where $\frac{{\mathrm{s}}_{\mathrm{rj}}^{+}}{{\mathrm{y}}_{\mathrm{rj}}}$ is standardized slack (ratio of slack to output), β_ro_ is intercept of r^th^ equation, β_rk_ is the coefficient of environment variable, k is the number of environmental variable, Z is the value of environmental variable, v_rj_ is random error, υ_rj_ is technical inefficiency, r (*r* = 1, 2,3, …, N) is the number of outputs, j (j = 1, 2,3, …, N) is the number of DMUs, k (k = 1, 2,3, …, N) is the number of environmental variables and o (o = 1, 2, 3, …, N) is the number of DMU under evaluation. In eq. (3) standardized slack regresses to environmental variables, random error and technical inefficiency.

To adjust the effect of random error and environmental conditions on technical efficiency, the following formula is used to modify all outputs according to similar environmental and random error condition (Eq. 4) [10].

4 $$ {y}_{rj}^a={y}_{rj}\left(1+{\upbeta}_{\mathrm{ro}}+\sum_{\mathrm{k}=1}^{\mathrm{K}}{\upbeta}_{\mathrm{rk}}{\mathrm{lnZ}}_{\mathrm{k}\mathrm{j}}+{\upnu}_{\mathrm{rj}}\right)={\mathrm{y}}_{\mathrm{rj}}\left(1+\frac{{\mathrm{s}}_{\mathrm{rj}}^{+}}{{\mathrm{y}}_{\mathrm{rj}}}-{\upupsilon}_{\mathrm{rj}}\right) $$

Where ${\mathrm{y}}_{\mathrm{rj}}^{\mathrm{a}}$ is the adjusted output,, y_rj_is the original output, β_ro_ is intercept of r^th^ equation, Z is the value of the environmental variable, *ν*_*rj*_ is random error, $\frac{{\mathrm{s}}_{\mathrm{rj}}^{+}}{{\mathrm{y}}_{\mathrm{rj}}}$ is standardized slack (ratio of slack to output), υ_rj_ is technical inefficiency of output, r (r = 1, 2,3, …, N) is the number of outputs, j (j = 1, 2,3, …, N) is the number of DMUs, k (k = 1, 2,3, …, N) is the number of environmental variables and o (o = 1, 2, 3, …, N) is the number of DMU under evaluation.

In stage three, the adjusted outputs were used in BBC-O model again. The Results of this stage showed pure technical efficiency.

In addition to exploring the most effective input variable on police efficiency, sensitivity analysis was also conducted through removing each variable in model and measuring efficiency score changes.

### Study setting and applied variables

This study was conducted using RAHVAR data from March 20, 2013 to March 20, 2014, according to Iranian calendar. Unit of analysis (DMU) was the rural police stations of all country. Put briefly, RAHVAR is a vice chancellor of Iran’s police (NAJA) at national level. It has sub directories in every province which are divided into two directories. One directory is responsible for traffic supervision in urban area and the other is about rural area that perform its tasks in police stations placed alongside the main roads.

Selection of Input and variables were based on the review of related studies and availability of valid data. Those variables were collected in a review article [21].

Input variables used in model were the number of police, patrol cars, police motorcycles, patrol teams, drivers penalized and punished, speed cameras and score of educational and cultural activity of police. The score of educational and cultural activity was derived according to annual evaluation of the send reports of provinces to RAHVAR. The assigned score was ranged 0–100.

The output variables were the number of crashes lead to damage, injury or death, separately. It is notable that if more than one outcome was occurred in a crash, the worst outcome would be considered. To exclude the effect of pre-hospital facilities on death rates, only the number of occurred deaths on crash scene was applied.

The environmental variables were in three main categories. They were the road infrastructure category; length of freeway/highway, length of one-way main roads, length of by-way roads, ratio of main roads to the total roads, the socioeconomic and demographic category; literacy rate, population of young people (aged 18–24), the number of household owning motorcycle, ratio of household owning car and traffic volume category; traffic count on main road (per minutes) in every traffic police jurisdiction.

### Data collection

Main data was obtained from RAHVAR. Speed camera variable obtained from road maintenance & transportation organization. Demographic and socioeconomic variables derived from national census information in 2011 and traffic count on main roads was measured with inductive loops installed across the main roads. They were downloaded from maintenance & transportation organization website in Excel format [URL: http://www.rmto.ir/Pages/TransportationCounter.aspx]. It is notable that the annual average of traffic count on the main road for every jurisdiction of police station was accounted.

The RAHVAR data was checked by a traffic officer.

### Model of analysis

The three-stage BCC-O model was used to measure the pure technical efficiency score of traffic police stations. In the first stage, BCC-O model was applied using the original input and output data. In the second stage, standardized slacks as the dependent variables were used in SFA model. In this stage, the effect of environmental variables and random error on dependent variables were distinguished. Using eq. (4), the original outputs were adjusted and used in stage three. In stage three, BCC-O model was conducted using adjusted output and original input data.

The DEA and SFA analysis were conducted with DEAP version 2.1 and STATA software version 11.

## Results

The data from 24 out of 31 provinces of Iran plus east region of Tehran province were collected. The data from Esfahan province with 15 stations was wrong; therefore, it was excluded. In addition, the data of 6 police stations because of missing data were excluded. In total, the information of 198 rural traffic police stations were analyzed that accounted for more than 74% of Iranian provinces. Razavi Khorasan province with 22 police stations and Yazd, Qom and North Khorasan provinces with 4 police stations had the most and the least stations respectively.

In total, 49,042, 36,608 and 5692 damage, injury and fatal accidents with means (standard deviation) of 247.6 (258.4), 184.9 (176.9) and 28.7 (19.5) occurred, respectively during March 20, 2013 to March 20, 2014. Other details are presented in Table 1.

**Table 1:** Descriptive characteristics of output variables

Output variables	Mean	SD	Minimum	Maximum
Number of damaged accidents^*^	247.7^a^	258.4	4.0	2181.0
Number of injury accidents^**^	184.9	176.9	3.0	1163.0
Number of fatal accidents^***^	28.7	19.5	2.0	99.0

^a^means on average, there have been 247.7 damaged accidents without any injury and death in every police station jurisdiction during a year

*Any accidents without any injury or death occurrence

**Any accidents that lead to one or more injury without any death

**Any accidents that lead to at least one death

Table 2 shows descriptive characteristics of input variables. In summary, on average (standard deviation) available resources for every police stations were 5.9 (3.0) patrol teams, 0.5% (0.2) proportion of total police man power (Based on Iran’s military regulations, reporting the exact number of military man powers is not allowed; therefore, the percentage of man power for every police stations was reported), 7.5 (2.9) patrol cars, 0.5 (1.3) motorcycles, 77,279.1 (46,794.7) penalties, 90.9 (2.8) cultural and educational activity score, 0.7 (2.4) speed cameras.

**Table 2:** Descriptive characteristics of input variables

Input variables	Mean	SD	Minimum	Maximum
Patrol team (number)	5.9	3.0	0,0	18.0
Manpower (percentage)	0.5	0.2	0.1	1.4
Patrol car (number)	7.5	2.9	2.0	18.0
Motorcycle (number)	0.5	1.3	0.0	8.0
Penalties and punishments (number)	77,279.1	46,794.7	7841.0	271,570.0
Cultural and educational (score)	90.9	2.8	84.0	95.8
Speed camera (number)	0.7	2.4	0.0	24.0

The mean (standard deviation) of environmental variables was 40.9 (57.3) km freeway/ highway, 24.9 (46.9) km one-way main roads, 104.1 (103.5) km two-way main roads, 382.3 (466.7) km by way, 0.84 (0.06) literacy rate, 14,344.8 (13,342.9) households owning motorcycle, 37.3 (9.1) percentage of household owning car, 38,936.4 (43,524.6) young people population, 6.4 (7.8) traffic count on main road per minutes, 19.8 (22.3) ratio of main roads to total roads. Other details are shown in Table 3.

**Table 3:** Descriptive characteristics of environmental variables

Environmental variables	Mean	SD	Minimum	Maximum
Length of freeway/ highway (km)	40.9	57.3	0.0	380.0
Length on one - way main roads (km)	24.9	46.9	0.0	300.0
Length of two - way main roads (km)	104.1	103.5	0.0	609.0
Length of by way roads (km)	382.3	466.7	0.0	2688.0
Literacy rate (percentage)	0.8	0.06	0.6	0.9
Number of household owning motorcycle	14,344.82	13,342.9	341.0	100,837.0
Percentage of household owning car	37.3	9.1	10,8	61.1
Population of young people (person)	38,936.4	43,524.6	1217.2	371,702.3
Mean of traffic count (per minute)	6.4	7.8	0.6	74.9
Proportion length of main roads to the total roads (percentage)	19.8	22,3	0.0	100.0

To show relationship of input and output variables the Pearson’s correlation coefficient between them were measured. It is shown in Table 4.

**Table 4:** Correlation matrix of input and output variables

Input variables	Crash	Injury	Death
Patrol team	0.27^***^	0.34^***^	0.35^***^
Manpower	0.24^***^	0.11	0.44^***^
Patrol car	0.42^***^	0.33^***^	0.25^***^
Motorcycle	0.06	0.17^*^	0.23^**^
Penalties and punishments	0.23^***^	0.22^**^	0.40^**^
Cultural and educational score	0.05	0.01	−0.00
Speed camera	0.23^**^	−0.02	0.05

^*^ *P* < 0.05, ^**^ *P* < 0.01, ^***^ *P* < 0.001

Before analysis, the data was checked. Since there were cases with zero values, to avoid error with DEA analysis, some positive values were added to data.

Based on the first stage BCC-O analysis, the mean (standard deviation) of technical efficiency scores was 0.92 (0.09). Among 198 traffic police stations, 30 (15%) had efficiency score of one. The others with efficiency score less than one ranged 0.38–0.99.

Before conducting stage two analysis with SFA model, the required assumptions of model were checked. One of them was half normal or truncated normal residual errors of OLS regression using standardized slack ratio as dependent variable and natural logarithm of environmental variable as independent variables. The results showed truncated normal distribution. Another assumption was negative skewness of residual error of OLS model. Since this assumption was not justified, to correct the wrong skewness, Feng Qu et al.’s proposed method (called finite sample correction method) was used. This method was used with k = 0.2 [20].

According to the second stage analysis, there were significant positive relation between one-way main road, by road, traffic count and the household owning number of motorcyclists and police inefficiency (*P* < 0.05). The most important relation was about the number of household owning motorcyclists. The length of freeway/ highway and literacy rate variables had significant negative relation with police inefficiency. According to H_0_ hypothesis test, there were not significant difference between police stations in efficiency score, after excluding the effects of environment and random error effects.

Other details are presented in Table 5.

**Table 5:** Results of SFA analysis

Natural logarithm of variables	Coefficient correlation	Standard error	CI (0.95)	
Freeway/highway	- 0.04^**^	0.01	−0.07	−0.02
One-way main roads	0.03^*^	0.01	0.01	0.05
Two-way main roads	Due to correlation with One-way main road it has been excluded.			
By roads	0.03^*^	0.01	0.01	0.05
Ratio of main road to total roads	0.03	0.01	−0.01	0.06
Young population	0.01	0.02	−0.04	0.04
Traffic count	0.19^**^	0.02	0.16	0.23
Literacy rate	−0.79^*^	0.37	−1.52	−0.05
Number of households owning motorcycle	0.08^**^	0.02	0.05	0.12
Proportion of household owning car	0.13	0.10	−0.06	0.33
Constant coefficient	1.93	1.39	−0.80	4.66
Mu	−2.07	145.55	−287.35	283.21
Ln sig^2^	−3.01	3.04	−8.98	2.96
Ilgtgamma	−2.65	46.09	−92.99	87.70
Sigma^2^	0.05	0.15	0.01	19.25
Gamma	0.06	2.84	4.10e – 41	1
Sigma *υ* ^2^	0.01	0.15	−0.29	0.30
Sigma *ν* ^2^	0.04	0.01	0.04	0.05

^*^ *P* < 0.05, ^**^ *P* < 0.01, ^***^ *P* < 0.001

Then, the outputs were adjusted according to environmental variables and random error. In stage three, the adjusted outputs were used in BCC-O model, again.

**Fig. 1:**
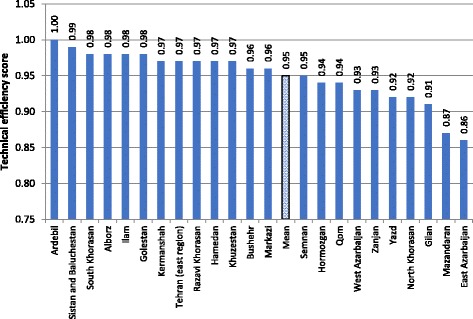
The average efficiency score of studied provinces

The efficiency score in this stage increased to 0.95. Similar to stage one, 30 units (15%) of police stations were efficient. On average, the police stations of Ardebil province had the highest efficiency score of 1 and East Azarbaijan had the lowest efficiency score of 0.86. It is noteworthy that the average of province score does not imply performance of headquarter of every province. To do this, two-level DEA model is required. There were not notable differences between studied provinces and there were not any geographical patterns. Figure 1 shows the average of efficiency score of police stations for the studied provinces.

**Fig. 2:**
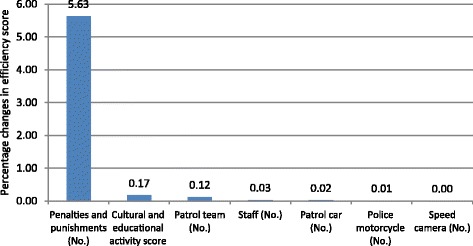
Sensitivity analysis results of input variables

The results of sensitivity analysis showed, among input variables, the number of penalties and punishments with 5.63% absolute changes had the most effect on police efficiency score. Figure 2 shows the results of sensitivity analysis of every input variable.

## Discussion

Resource limitation is an obstacle to implement some required intervention to reduce RTIs. This problem is more important in low and middle-income countries. Even in high income countries this limitation may induce governments to set RTIs targets on lower level. For instance, according to a survey in 1999 in Norway, continuing present measures will prevent 34 deaths out of 306 during 2002 to 2011. While with implementing all cost-benefit measures such decrease could reach 183 deaths (49% of annual present deaths). For Sweden it was 38% [14]. Increasing efficiency alongside selecting effective measures could be helpful in this issue.

Traffic police as an administrator of law enforcement has important role in reducing RTIs. Well performance of this organization not only reduces RTIs but also saves resources.

The relative efficiency of Iran’s traffic police was measured in this study with three-stage DEA model for the first time. This is conducted with excluding the effects of seven environmental variables and random errors from technical efficiency score. The input and output variables were police resources with 7 variables and 3 outcomes due to a crash. The analysis results showed average efficiency score of 0.95 of which 15% had perfect score without any significant difference. This indicates potential opportunity to reduce RTIs with present resource to 5%. Among studied provinces, Ardebil province with the mean of 1 had the most efficient police stations and East Azarbaijan had the least efficient ones.

For the first time, instead of setting a general target for the whole country, a short-term (one year) target was provided for each police station in Iran. What’s more, a rational comparison was made between the police stations. This induces incentive and competition for policy makers and managers to reduce RTIs.

The results of environmental analysis showed positive relation among one-way main road, by road, traffic count and the number of household owning motorcycles and police inefficiency. Significant reverse relation between the length of highway/freeway with the police inefficiencies indicates that the investment in road infrastructure not only reduces traffic accidents but also improves the performance of the police. In addition, it is necessary to adjust police strategy for area with more motorcycle and heavy traffic roads. It is also true for high season journey periods. Literacy rate as the proxy of cultural and behavioral status had positive relation with efficiency score. These findings confirmed the effect of human factor on RTIs.

Depending on the relative results of the DEA method, it is not possible to generalize and compare the findings with other studies. Other reason refers to using different variables in these studies compared to others [3, 13].

According to sensitivity analysis, the number of penalties and punishment was recognized as the most effective input on police efficiency. This finding is in concordance with D de Waard et al. study that showed on-view stopping offenders is more effective to reduce driving speed compared to mailing fines [11]. On-view stopping violating drivers increase apprehension level among potential violating drivers; as a result, it prevents committing risky behaviors. Iran has lower traffic safety culture and higher traffic violators [18, 19]. On-viewing police punishment alongside the roads in low safety context has more chance of offender exposures to this scene, compared to higher safety context. Therefore, the police intervention becomes more efficient in societies with low safety culture. The second effective variable was cultural and educational score. As this variable was not specific for every police station, it is doubtful to conclude about its effect. However, despite this limitation, the cultural and educational activity score could be as a proxy of managerial status of every province which affected police performance in rural areas. Higher score may indicate more rigid supervision on police stations by headquarters.

The least effective variables were the number of speed camera and police motorcycles. It is attributed to the high homogeneity of study samples. About 86% of the police stations did not have speed camera. It is true for the police motorcycle variable with 77%.

In this study, applying a comprehensive set of input and output variables with large sample size prepared acceptable condition for efficiency measurement. In case of inadequate sample size and high number of variables, the DEA model considers most DMUs efficient with the score of 1 by default; hence, the model losses its discrimination power [10]. Other issue refers to controlling non-discretional variables (environmental variables). To control the effect of non-discretional variables, different methods have been proposed. One of which is using specific DEA models that considers non-discretional variables as a parameter in model formula [10]. This method could not account random error in measurement. The other method is clustering similar DMUs according to environmental variables and applying each cluster, independently. Clustering DMUs leads to sample size reduction and decreasing discrimination power. Although there is super efficiency model to merge all cluster and obtain unique efficiency score [2], it is not possible to account for random error in efficiency measurement. As the strength of study, using three-stage model avoided sample size reduction while accounting for random error in efficiency measurement.

This study had some limitations. Using current official data instead of data gathering was one of them. Despite checking data with an expert officer, the validity of data could not be assured. Another limitation refers to applying cultural and educational activity score and the number of penalized and punished violated drivers. It seems these variables were intermediate variable between inputs and final outputs. Therefore, it was better to conduct two-stage DEA model. At the first stage, the efficiency score is measured using inputs and these variables as the outputs and at second stage, these outputs are applied as inputs and final outcomes as outputs. Since the cultural and educational activity score were taken from the province activity and not specific to every police station, to use two-stage model was abandoned.

Based on DEA extensions and models, further studies to measure allocative efficiency are recommended. Such model can determine the most efficient and cheapest traffic police input combination, especially in low and middle-income countries. To conduct such study, access to cost of input data is required. Other study which is recommended is exploring the trend of police efficiency and extracting efficiency due to technological changes from technical efficiency, using DEA-based Malmquist productivity index (MPI). It demands data collection during successive years. Taking the results of this study into account, almost all police stations had the efficiency scale of one. Therefore, finding the most productive scale size (MPSS) was not attainable. Further studies to check this finding is recommended.

## Conclusions

RTIs as the heath problem in Iran requires conducting different interventions and investing fund. Because of lower safety culture among Iranian drivers [18, 19], the role of traffic police is predominant. Therefore, monitoring and evaluating traffic police performance with increasing its efficiency not only increases resource efficiency but also induces RTIs reduction.

## Abbreviations

BCC-O: Banker, Charnes and Cooper Output oriented model
DEA: Data Envelopment Analysis
NAJA: Iranian Police
OLS: Ordinary Least Square
RAHVAR: Iranian Traffic Police
RTIs: Road Traffic Injuries
SFA: Stochastic Frontier Analysis
